# Transcriptional Regulation of Mouse Tissue-Resident Natural Killer Cell Development

**DOI:** 10.3389/fimmu.2020.00309

**Published:** 2020-02-25

**Authors:** Nuriban Valero-Pacheco, Aimee M. Beaulieu

**Affiliations:** ^1^Center for Immunity and Inflammation, New Jersey Medical School, Rutgers Biomedical and Health Sciences, Rutgers – The State University of New Jersey, Newark, NJ, United States; ^2^Department of Microbiology, Biochemistry, and Molecular Genetics, New Jersey Medical School, Rutgers Biomedical and Health Sciences, Rutgers – The State University of New Jersey, Newark, NJ, United States

**Keywords:** natural killer cells, tissue-resident NK cells, transcriptional regulation, transcription factors, group 1 innate lymphoid cells

## Abstract

Natural killer (NK) cells are cytotoxic innate lymphocytes that are well-known for their ability to kill infected or malignant cells. Beyond their roles in tumor surveillance and anti-pathogen defense, more recent studies have highlighted key roles for NK cells in a broad range of biological processes, including metabolic homeostasis, immunomodulation of T cells, contact hypersensitivity, and pregnancy. Consistent with the breadth and diversity of these functions, it is now appreciated that NK cells are a heterogeneous population, comprised of specialized and sometimes tissue-specific subsets with distinct phenotypes and effector functions. Indeed, in addition to the conventional NK cells (cNKs) that are abundant and have been well-studied in the blood and spleen, distinct subsets of tissue-resident NK cells (trNKs) and “helper” Group 1 innate lymphoid cells (ILC1s) have now been described in multiple organs and tissues, including the liver, uterus, thymus, adipose tissue, and skin, among others. The cNK, trNK, and/or helper ILC1 populations that co-exist in these various tissues exhibit both common and distinct developmental requirements, suggesting that a combination of lineage–, subset–, and tissue–specific differentiation processes may contribute to the unique functional properties of these various populations. Here, we provide an overview of the transcriptional regulatory pathways known to instruct the development and differentiation of cNK, trNK, and helper ILC1 populations in specific tissues in mice.

## Introduction

Natural killer (NK) cells are cytotoxic innate lymphocytes that were first identified in 1975 based on their capacity to spontaneously kill tumor cell lines without prior immunization (1, 2). Over the past 45 years, our understanding of NK cell biology has grown and evolved, and it is now clear that NK cells play important roles in diverse biological processes, ranging from tumor surveillance and anti-pathogen defense to metabolic disorders, inflammatory diseases, stem cell transplantation, neuronal pruning, and pregnancy (3–9).

The balance of evidence suggests that mouse NK cells and helper ILC1s are distinct lineages in mice, arising from separate lineage-committed progenitors under homeostatic conditions (10). They do, however, share extensive phenotypic and functional similarities, including expression of many markers historically associated with NK cells such as NKp46, robust production of interferon gamma (IFN-γ) upon activation, and expression of the T-box transcription factor, T-box expressed in T cells (T-bet) (10). In certain tissues and inflammatory settings, NK cells and helper ILC1s can possess such similar phenotypes that they have been difficult to distinguish, particularly in the absence of lineage-tracing experiments. These challenges, along with the historical lag in recognizing NK cells and helper ILC1s as distinct lineages and the fact that few truly lineage-discriminating markers have been described, have resulted in a confusing body of literature in which NK cells and helper ILC1s have not always been separately identified or consistently defined. For consistency within this review, we will discriminate mouse NK cells from mouse helper ILC1s on the basis of Eomesodermin (Eomes) expression, a common convention notwithstanding the field's limited understanding of conditions under which NK cells might lose, or helper ILC1s might gain, Eomes.

While most studies on NK cells have focused on population(s) abundant in the blood and spleen, now commonly referred to as conventional NK cells (cNKs), unique tissue-specific and/or tissue-resident NK cell (trNK) populations have recently been described in diverse tissues, including the uterus, thymus, intestine, adipose, skin, peritoneal cavity, and salivary, lacrimal, and mammary glands. As described below, many of these unique trNK populations exhibit distinct tissue-specific phenotypes, functions, and developmental requirements. In particular, recent studies have highlighted notable differences in the transcriptional regulation of trNK, cNK, and helper ILC1 development, suggesting that distinct differentiation processes support the unique functional properties of tissue-specific trNKs. Here, we review current literature on the transcriptional pathways known to control the development of various trNK populations in mice, with a particular focus on regulatory mechanisms that are unique to trNKs as compared to cNKs and helper ILC1s in each tissue.

## Overview of cNK Development in the Bone Marrow

The bone marrow is the primary, but not exclusive, site of cNK development in adults (11–13). Like T cells and B cells, cNKs develop from precursor populations with pan-lymphocyte potential—e.g., common lymphoid progenitors (CLPs) and lymphoid-primed multipotent progenitors (LMPPs)—via a stepwise differentiation process in which multi-lineage potential progressively diminishes as the NK cell fate becomes established (14, 15). Early innate lymphoid progenitors (EILPs) and alpha-lymphoid progenitors (αLPs) are among the earliest developmental intermediates capable of generating NK cell-committed NK progenitors (NKPs) and helper ILC-committed ILC precursors (ILCPs), but not T cell- or B cell-committed precursors (16–18). Mouse NKPs were originally reported to exist within a pool of Flt3^−^2B4^+^CD27^+^Id2^hi^IL-7Ra^+/−^ cells in the bone marrow that lacked all mature immune cell lineage markers (Lin^−^), including classical NK cell markers such as NKp46 (19–21). These included very early NKPs (e.g., pre-NKPs and pre-pro-NKPs) that lacked the IL-15 receptor β-chain, CD122, as well as more differentiated “refined” NKPs (rNKPs) that expressed CD122 and were thus responsive to IL-15, a cytokine known to critically regulate diverse aspects of cNK development and function (20, 21). NKPs were shown to give rise to immature NK cells (iNKs), which had acquired expression of the NK activing receptors NKp46 and, in some mouse strains, NK1.1 (19). [Of note, later lineage-tracing studies demonstrated that the markers originally used to identify NKPs, rNKPs, and iNKs in the bone marrow did not fully exclude all helper ILC lineage cells, especially helper ILC1s (22)]. Upregulation of CD49b, additional NK receptors (e.g., Ly49 receptors), and effector molecules such as perforin and granzymes mark the later stages of differentiation into mature NK cells (mNKs) (19–21). mNKs continue to mature in the bone marrow and peripheral tissues, a process marked by downregulation of CD27 and upregulation of CD11b, with CD27^+^CD11b^−^ cells being less mature (but more proliferative) and CD27^−^CD11b^+^ cells being most mature (23–25).

## Transcriptional Regulation of cNK Development in the Bone Marrow

cNK development is controlled by the sequential and coordinated activities of multiple transcriptional regulators. Among these are the transcription factors T cell factor 1 (TCF-1) and Nuclear factor interleukin-3 regulated (Nfil3), both of which are expressed at or prior to the NKP-ILCP developmental branch point and are important for proper cNK and helper ILC lineage differentiation (17, 18, 26–34). Mice lacking TCF-1 have fewer pre-NKPs, rNKPs, and mNKs in the bone marrow. And, although peripheral cNK numbers are only modestly impacted in non-chimeric TCF-1-deficient mice, they are severely reduced in a competitive mixed bone marrow chimera environment (18, 34). Notably, TCF-1-deficient NK cells have an unusual hypermature but pro-apopotic phenotype linked to granzyme B overexpression, suggesting that TCF-1 controls cNK development by modulating the timing of maturation and effector gene expression (34).

Nfil3-deficient mice also have severe and early defects in NK cell development, reflected in a near-complete loss of cNKs in the periphery and significantly reduced numbers of NKPs, iNKs, and mNKs in the bone marrow (17, 26, 27, 31, 33). The requirement for Nfil3 appears to be restricted to the earliest stages of cNK development, as loss of Nfil3 at or after the iNK stage has little impact on cNK numbers or function (35). Nfil3 itself regulates expression of several other transcription factors important for NK differentiation and maturation, including Inhibitor of DNA binding 2 (Id2) and Eomes (discussed below) (26, 31, 32). Id2, which acts to inhibit E-box family proteins that support B and T cell differentiation, is indispensable for cNK development. Id2-deficiency leads to a severe reduction in the peripheral cNK compartment, owing to its critical roles in promoting cNK maturation, effector functionality, and sensitivity to IL-15 signaling (36–39).

Like Nfil3, the transcription factors, ETS proto-oncogene 1 (Ets1) and Signal transducer and activator of transcription 5 (Stat5), the histone H2A deubiquitinase, Myb-like, SWIRM and MPN domains 1 (Mysm1), and the long non-coding RNA (lncRNA), RNA-demarcated regulatory region of Id2 (*Rroid*) also critically regulate cNK development and are important for maintaining proper Id2 expression in differentiating cNKs (40–43). Genetic deficiencies in Ets1, Mysm1, or *Rroid* all impair maturation of bone marrow cNKs, resulting in fewer, less mature, and less functional cNKs in the periphery (42, 43). Similarly, mature peripheral cNKs are severely reduced in mice lacking Stat5b, and to a lesser extent Stat5a (43–45), and Stat5 tetramerization was recently shown to support cNK maturation in the bone marrow and spleen (46).

Other important regulators of cNK development include the T-box family transcription factors, T-bet and Eomes. Deficiencies in either factor result in impaired cNK maturation in the bone marrow, leading to fewer and less mature cNKs in the periphery (47–52). T-bet in particular is important for modulating proliferation and supporting survival in maturing cNKs (47). Eomes and T-bet have both unique and overlapping functions in developing cNKs. For example, T-bet-deficiency only moderately impacts peripheral cNK numbers, and has little impact on bone marrow cNK abundance, whereas Eomes-deficiency substantially reduces both bone marrow and peripheral cNK numbers (50, 52). Moreover, compound deficiencies in both factors are far more deleterious than deficiencies in either factor alone, resulting in a near-complete loss of cNKs in the bone marrow and peripheral organs (48, 50).

Additional transcription factors known to regulate later stages of cNK cell differentiation and maturation include Kruppel-like factor 2 (KLF2), GATA binding protein 3 (Gata-3), Runt-related transcription factor 3 (Runx3), and Zinc-finger E homeobox-binding 2 (Zeb2). Similar to T-bet, KLF2 restricts abnormal proliferation and supports survival in maturing cNKs, and KLF2-deficiency reduces the number of mature cNKs in the periphery (53). Gata-3 helps sustain Id2, T-bet, and Nfil3 expression in maturing cNKs, and cNKs lacking Gata-3 exhibit defects in maturation and bone marrow egress (54). Similarly, Runx3 promotes later stages of cNK maturation, possibly through cooperative regulation with T-box and Ets family transcription factors, and cell-specific deletion of Runx3 or its co-regulator Cbf-β leads to a reduction in the peripheral cNK compartment (55, 56). And finally, Zeb2 has been shown to act downstream of T-bet to critically regulate the maturation, survival, and egress of mature cNKs from the bone marrow. Mice lacking Zeb2 have more immature cNKs in the bone marrow, and fewer mature cNKs in the periphery (57).

## Development of Tissue-Specific or Tissue-Resident NK Cells and Helper ILC1s

### Liver

In addition to circulating CD49a^−^CD49b^+^Eomes^+^ cNKs, the liver harbors a unique population of CD49a^+^CD49b^−^Eomes^−^ ILC1s that are tissue-resident in parabiotic mice (58, 59). [Different groups refer to these tissue-resident cells as either liver trNKs or liver ILC1s; here, we will use the ILC1 designation since these cells are Eomes^−^]. Liver ILC1s reside in the liver sinusoids and have been shown to mediate memory-like immune responses in models of contact hypersensitivity (CHS) and viral infection (59–62).

Phenotypically, liver ILC1s resemble immature cNKs in having low or no expression of killer cell lectin-like receptor G1 (KLRG1), CD11b, CD122, and Ly49 receptors such as Ly49A, Ly49D, Ly49G2, and Ly49H (50, 51, 63, 64). However, liver ILC1s are transcriptomically distinct from both immature and mature cNKs and exhibit an activated phenotype at steady state, characterized by high expression of CD69, CD44, and CD160, and low expression of CD62L (also known as L-selectin) (51, 59, 64, 65). They also express high levels of tumor necrosis factor (TNF)-related apoptosis-inducing ligand (TRAIL) and CD127, as well as chemokine receptors such as CXCR3 and CXCR6 that support residence in the liver sinusoids (50, 51, 59, 64, 66, 67). Although activated liver ILC1s retain cytotoxic functionality against target cells, they differ from cNKs in their higher production of TNF-α, IL-2, and granulocyte-macrophage colony-stimulating factor (GM-CSF), their preferential expression of granzyme C instead of granzyme B, and their reduced expression of perforin (51, 59, 64, 65). Liver ILC1s also express molecules involved in immune regulation, including PD-L1, LAG3, CD39, and CD73, and were recently shown to inhibit T cell function via the PD-1–PD-L1 axis (68).

The unique phenotype, function, and transcriptome of liver ILC1s, as well as the finding that they do not give rise to Eomes^+^ NK cells following adoptive transfer into intact (un-irradiated) hosts (51), support their identity as a distinct lineage. Consistent with this, liver ILC1s and cNKs exhibit both common and distinct developmental requirements. Commonalities include their shared dependence on IL-15 but not IL-7 signaling for development, notwithstanding constitutive expression of CD127 by liver ILC1s (51). Additionally, both liver ILC1s and liver cNKs require TCF-1, Gata-3, Runx3 and its co-factor Cbf-β, and T-bet for development, although liver ILC1s are more severely impacted by T-bet-deficiency than liver cNKs (18, 50–52, 54, 56, 64).

Distinct developmental requirements include findings that liver ILC1s require Promyelocytic leukemia zinc finger (PLZF) and the Aryl hydrocarbon receptor (AhR) for development, two transcription factors that are dispensable for cNK development (22, 69). Loss of PLZF significantly impairs liver ILC1 development, similar to its impact on many other helper ILC sublineages (22). Likewise, mice lacking AhR have reduced numbers of liver ILC1s, but normal numbers of liver cNKs, owing to a role for AhR in limiting turnover and susceptibility to cytokine-induced death in liver ILC1s (69).

Nfil3 has been reported as dispensable for liver ILC1, but essential for liver cNK, development in some studies (32, 64, 70), although Nfil3-deficient liver ILC1s were recently reported to be competitively disadvantaged in mixed bone marrow chimeric mice (52). Liver ILC1s also do not require KLF2 or Eomes for development, unlike liver cNKs (50, 52, 53).

Development of liver ILC1s also uniquely depends on Homolog of Blimp-1 (Hobit), a transcription factor that supports T cell tissue-residency, in part by suppressing genes involved in tissue egress (71). Notably, although liver ILC1s are severely reduced in Hobit-deficient mice, NK and helper ILC1 populations in other organs remain largely unaffected, suggesting that Hobit is a liver-specific regulatory factor for ILC1s (52, 71).

### Uterus

NK cells are one of the most abundant immune cell types in the uterus at steady state and during early- and mid-pregnancy. Uterine NK cells have been shown to regulate diverse processes in female reproductive biology, and are particularly critical for remodeling of the uterine vasculature during early pregnancy [Reviewed in Croy et al. (72)]. At least three distinct NK and/or ILC1 populations exist in the uterus, each with unique transcriptional signatures: CD49a^−^CD49b^+^Eomes^+^ cNKs, tissue-resident CD49a^+^CD49b^−^Eomes^+^ trNKs, and CD49a^+^CD49b^−^Eomes^−^ helper ILC1s (73–76). The frequency and distribution of these populations vary with respect to sexual maturity and reproductive state (73, 75–77). Uterine helper ILC1s are most abundant in pre-pubertal mice, and exhibit preferential expansion during repeat pregnancies, whereas sexual maturity is associated with a decrease in the frequency of helper ILC1s and an increase in trNKs and cNKs (75). During pregnancy, trNKs further proliferate *in situ* and remain abundant throughout early decidualization (76). Placentation and mid-pregnancy are associated with a decrease in trNK cells and an increase in cNKs, with the latter comprising the majority through birth and weaning (75).

The origin and developmental requirements of uterine NK cells are only partially understood, although some appear to arise from *in situ* progenitors and others from recruited cells (77–79). Uterine trNKs develop normally in athymic mice and do not express CD127 at steady state, indicating that their development is distinct from thymic NK cells (discussed below) (64). Uterine NK cell development has been reported to be IL-15-dependent, although whether uterine trNKs, cNKs, and helper ILC1s all require IL-15 signaling to the same extent remains to be determined (80, 81).

With respect to transcriptional regulation of uterine NK and helper ILC1 development, Nfil3 has been implicated in some but not all studies, likely owing to differences in strategies used to identify cNKs, trNKs, and helper ILC1 populations in the uterus. Nfil3-deficiency is consistently associated with significantly reduced numbers of cNKs in the uteri of both virgin and pregnant mice (64, 74, 82). However, Nfil3-deficient mice were reported to have normal numbers of uterine CD49a^+^CD49b^−^ cells – a population that includes trNKs and helper ILC1s—in one study (64), but reduced trNK and normal helper ILC1 numbers in another study (74). The latter study showed that trNKs in Nfil3-deficient mice were able to expand in response to pregnancy, although defects in decidual vascularization and placentation persisted, possibly due to the persisting deficit in uterine cNKs and Group 2 ILCs (ILC2s) (74, 82).

In addition to Nfil3, T-bet and Runx3 have also been evaluated for their impact on uterine NK cell development. T-bet-deficiency does not alter the overall abundance of uterine CD49a^+^CD49b^−^ cells, although differential effects on trNK vs. helper ILC1s have not been assessed (64). In contrast, implantation site NK cells were strikingly absent in pregnant *Runx3*^−/−^ mice, although the requirement for Runx3 in development of specific uterine NK or ILC1 subsets remains unknown (55, 83).

### Thymus

The thymus harbors a unique population of Gata-3^+^ NK cells with a CD127^+^CD11b^lo^CD69^hi^CD49b^+^CD49a^−^ surface phenotype and low expression of Ly49 receptors (84, 85). Functionally, these thymic NK cells (tNKs) are less cytotoxic but produce more IFN-γ than splenic cNKs, and are similar to liver trNKs in their ability to produce TNF-α and GM-CSF (84). Unlike T cells, tNKs do not require Notch signaling for development and do not develop from T cell-committed progenitor cells (86, 87). They can, however, develop from NKPs in the fetal thymus (88), and from early double-negative (DN) 1 and DN2 thymocyte precursors (70, 89–91).

The molecular requirements for tNK development are unique. Unlike cNKs and many other trNK populations, tNK cells require both IL-7 and IL-15 signaling for development (84, 92). Moreover, genetic deficiencies that disrupt tetramerization of Stat5, which signals downstream of both IL-7 and IL-15, reduce overall tNK numbers (46). tNK cell development is also strictly dependent on Gata-3, mirroring the requirement for Gata-3 in T cell development past the DN2 stage (84, 93). tNK development has been reported as Nfil3-dependent in some (32, 85) but not all (70) studies, possibly due to differences in mouse strains and gating strategies across studies.

Ets1 and Id2 also play important roles in tNK development. Ets1-deficient mice (on a *Rag1*^−/−^ background) harbor fewer tNKs overall, and those present have a CD11b^hi^KLRG1^hi^CD27^lo^ phenotype typically associated with mature cNKs (85). Conversely, Id2-deficient *Rag1*^−/−^ mice have normal numbers of tNKs, but these have an abnormal CD27^hi^CD11b^−^ phenotype reminiscent of immature cNKs (85).

Both Mysm1 and T-bet are dispensable for tNK development, although tNK cells do express T-bet (42, 85). They also express Bcl11b, a zinc finger transcription factor that is essential for T cell development (94). Bcl11b–deficient thymocytes have been shown to acquire an NK cell-like phenotype, although whether these cells represent *bona fide* tNKs remains unclear (94–96).

### Salivary Glands

The salivary glands (SG) contain several tissue-resident NK and helper ILC1 populations with unique phenotypes and functions (97–101). Among these, NK lineage cells represent ~80–90% and helper ILC1s ~10–20% of the total pool, based on lineage tracing studies involving PLZF–reporter/fate mapping mice and patterns of Eomes expression (101). Notably, both SG helper ILC1s and the majority of SG NKs exhibit long-term tissue-residency in parabiotic mice, and peripheral cNKs are not recruited to the SG even during viral infection, suggesting that trNKs constitute a sizeable fraction of the NK lineage compartment (97, 99).

SG NK cells have a distinct surface phenotype, with most co-expressing both CD49a and CD49b, although small populations of CD49a^−^CD49b^+^ cNK-like cells and CD49a^+^CD49b^−^ cells are also present (101). SG helper ILC1s are predominantly CD49a^+^CD49b^+^ or CD49a^+^CD49b^−^ (101). At steady state, SG NKs exhibit low or no expression of CD27, CD43, CD127, and KLRG1, but express high levels of CD69 and CD44 (97, 98). ~40% also express CD103 (also known as integrin alpha E), a marker often associated with tissue residency (98).

Functionally, SG NK cells are poor producers of IFN-γ and degranulate less than splenic cNKs (97, 98). However, some SG NK cells do express TRAIL. Notably, SG NKs were shown to cull activated SG CD4^+^ T cells in a TRAIL-dependent manner during chronic viral infection (98, 101, 102). This activity was important for limiting autoimmune-like tissue destruction, suggesting that SG NK cells may be critical modulators of pathogenic T cell responses in the SG (102).

SG NK cells are critically dependent on a non-canonical Smad4-independent TGF-β signaling pathway for development or maintenance (100). Mechanistically, TGF-β is thought to act by modulating Eomes expression—CD49a^+^ SG NKs are Eomes^mid^ in contrast to the CD49a^−^ Eomes^hi^ cNK-like population in the SG—and by enhancing expression of other factors that support NK cell survival (100). In line with this, disruption of TGF-β signaling impairs both the abundance and distinct surface phenotype of SG NK cells (100).

SG NK cell development was initially reported to be Nfil3-independent (98), but later studies involving PLZF–reporter/fate-mapping mice and mixed bone marrow chimeric mice demonstrated that the majority of SG NK cells develop in an Nfil3-dependent manner, and only a minor fraction are Nfil3-independent (52, 101). Although the Nfil3-dependent and -independent populations have similar surface phenotypes and are functionally hyporesponsive when in the SG, these features are specifically reversible in the Nfil3-dependent subset following transfer into the spleen or liver (101). Thus, tissue-specific signals likely instruct the unique phenotype of SG NK cells.

All SG NK and helper ILC1 populations are T-bet^hi^ (98, 100, 101). Although experiments in mixed bone marrow chimeric mice suggested that T-bet is important for SG ILC1, and to a lesser extent SG NK, development in a competitive setting (52), non-chimeric *Tbx21*^−/−^ mice (T-bet is encoded by *Tbx21*) have a relatively intact SG NK compartment (100). Eomes^−^ SG helper ILC1s do not require Eomes for development (52). However, the role of Eomes in SG NK development is surprisingly nuanced. As mentioned above, CD49a^−^ SG cNKs are Eomes^hi^ and CD49a^+^ SG trNKs are Eomes^mid^ owing to TGF-β-mediated restriction of Eomes expression in the latter subset (100). Unexpectedly, cell-specific deletion of Eomes in non-chimeric mice does not alter the overall abundance of total SG NKs, but rather reduces the fraction that expresses CD49b and actually enhances the distinctive surface phenotype of SG NKs (100). These findings highlight an unusual modulatory role for Eomes in SG NK development or maintenance, which contrasts with the generally strict requirement for Eomes in cNK development.

Other transcriptional regulators that have been evaluated for roles in SG NK and helper ILC1 development include Hobit and the lncRNA *Rroid*, as well as Runx3 and its co-factor Cbf-β. Both SG helper ILC1s and CD49b^+^ SG NKs develop independently of Hobit, a feature that distinguishes them from liver ILC1s (52). Similarly, the SG compartment is largely unperturbed in mice lacking the lncRNA *Rroid* (43). In contrast, cell-specific deletion of Runx3 or Cbf-β results in a significant reduction in the total SG compartment, underscoring a key role for the Runx pathway in SG NK and/or helper ILC1 development (56).

### Intestines

Several IFN-γ-producing NK and helper ILC1 populations have been identified in the intestinal mucosa of mice. These include Lin^−^CD160^+^NK1.1^+^NKp46^+^ intraepithelial lymphocytes (IEL), comprised of both Eomes^+^ NK cells and Eomes^−^ helper ILC1s, which have been implicated in colitis-associated inflammation (103, 104). Additionally, the small intestine lamina propria (siLP) harbors both CD49b^+^Eomes^+^ cNK-like cells and CD49a^+^Eomes^+^ trNK-like cells, in addition to a population of CD49a^+^CD49b^−^Eomes^−^ helper ILC1s that contribute to defense against certain enteric pathogens (99, 104, 105). Phenotypically, siLP helper ILC1s are CD127^+^CD62L^lo^CD69^hi^CD44^hi^ cells that exhibit low or no expression of CD11b and most Ly49 receptors (104), but variously express CCR9, CXCR3, and CXCR6, chemokine receptors associated with lymphocyte homing to tissues (104, 105). Functionally, siLP helper ILC1s produce more IFN-γ, TNF-α, and GM-CSF than cNKs, a phenotype that is reminiscent of liver ILC1s, but have low expression of cytotoxicity-associated molecules such as granzyme B, perforin, and CD107a (104, 105). Parabiosis studies demonstrated that siLP helper ILC1s, and possibly a portion of siLP NK cells, are genuine tissue-resident cells (99, 105).

With respect to developmental requirements, both siLP NKs and siLP helper ILC1s are significantly reduced in *Il15*^−/−^, but not *Il7Ra*^−/−^, mice, indicating that IL-15 signaling is critical for the development and/or maintenance of both populations (104). In contrast to findings in the siLP, the overall IEL compartment is only modestly reduced in *Il15R*α^−/−^ mice, although the differential impact on NK cells vs. helper ILC1s was not evaluated (103). Nevertheless, partial gene deficiencies in *Stat5a* and/or *Stat5b* result in fewer helper ILC1s in both the IEL and siLP compartments, as well as fewer IEL NK cells (45).

In addition to Stat5, several other transcriptional regulators are known to impact intestinal NK and/or helper ILC1 development. For example, siLP helper ILC1s require T-bet and Gata-3 for development or maintenance, but not Eomes, the lncRNA *Rroid*, or Retinoid-related orphan receptor gamma t (RORγt) (43, 104, 105). In contrast, siLP NKs require Eomes but not T-bet (104, 105). The bulk IEL population is significantly reduced in mice lacking T-bet, Runx3, or Cbf-β, although the extent to which these factors differentially impact the Eomes^+^ NK vs. Eomes^−^ ILC1 fractions remains unclear (56, 103). In contrast, both AhR and RORγt are dispensable for IEL NK cell and/or helper ILC1 development (103). And finally, Nfil3 appears to be required for all described intestinal populations: siLP NK cells, siLP helper ILC1s, and the IEL population(s) (103–105).

### Adipose Tissue

IFN-γ-producing NK and helper ILC1 populations in the adipose tissue (AT) have been shown to contribute to obesity-related metabolic dysfunction, in part by promoting the differentiation of inflammatory M1 macrophages (106–110). The AT NK compartment includes sizeable populations of CD49a^−^CD49b^+^Eomes^+^ cNKs, and CD49a^−^CD49b^−^Eomes^+^ and CD49a^+^CD49b^−^Eomes^+^ NKs that have been called immature NK cells by some groups (109, 110). Eomes^−^ helper ILC1s are also abundant in the AT, the majority of which are CD49a^+^CD49b^−^ (109, 110). Parabiosis studies indicate that AT helper ILC1s and many of the CD49b^−^ NKs are genuine tissue-resident cells, whereas mature cNKs in the AT are non-resident (109, 110).

The CD49b^−^ trNK populations in the AT have unique surface phenotypes. Most are CD90^hi^CD69^hi^ and variably express many cNK-associated molecules (e.g., CD11b, KLRG1, Ly49D, and Ly49H) but not CD127 (109, 110). All NK and helper ILC1 populations in the AT are TRAIL^−^. CD49b^−^ trNKs and helper ILC1s express lower levels of granzyme B than mature cNKs in the AT (110). In addition, AT helper ILC1s produce more IFN-γ than AT cNK cells, possibly reflecting a central role in AT macrophage polarization and/or metabolic dysfunction (107, 109, 110).

Important transcriptional regulators of AT NK cells and ILC1s include Nfil3, which is critical for development of all cNKs, trNKs, and helper ILC1s in the AT (52, 109, 110). The roles of T-bet and Eomes, on the other hand, appear to be subset-specific. T-bet-deficiency selectively affects AT cNKs and helper ILC1s, particularly in mixed bone marrow chimeras and in *Rag2*^−/−^ mice, but has little impact on the CD49b^−^ trNK compartment (52, 109, 110). In contrast, Eomes is strictly required for development of cNKs but not helper ILC1s in the AT (its role in CD49b^−^ trNK development remains unclear) (52). And lastly, both AT cNKs and helper ILC1s develop in a Hobit-independent manner (52).

### Skin

In addition to CD49a^−^CD49b^+^Eomes^+^ cNKs, mouse skin harbors a distinct population of CD49a^+^CD49b^−^Eomes^lo/−^ cells that are CD69^hi^CD127^−^ and are largely tissue-resident in parabiotic mice (64). This tissue-resident population has been referred to as a trNK subset, although its lack of Eomes expression suggests a helper ILC1 identity. Both skin cNKs and the skin-resident ILC1s require Runx3 and its co-factor Cbf-β for development (56). The skin-resident ILC1s are similar to liver-resident cells in requiring T-bet and IL-15, but not Nfil3, for development (64). Notably, liver- and skin-resident populations not only share many developmental requirements, but they also appear to cooperate in inter-organ immune responses involving the skin and liver. For example, liver ILC1s can mediate hapten-specific CHS memory responses in the skin, and hapten-specific memory ILC1s in the skin-draining lymph node were recently shown to migrate to and reside in the liver (59–61, 64, 111, 112). Altogether these findings point to a unique relationship between the liver- and skin-resident populations that remains to be fully defined.

### Peritoneum

The peritoneal cavity (PC) contains both CD49a^−^CD49b^+^Eomes^+^ cNKs and PC-resident CD49a^+^CD49b^−^Eomes^−^ helper ILC1s (52, 64, 113). At steady state, PC helper ILC1s are CD200r1^+^CD61^+^CD27^+^ cells that lack expression of Ly49H, CD11b, CD69, and CD103 (52, 113). In response to viral challenge in the peritoneum, PC helper ILC1s produce more IFN-γ than PC cNKs (52). Transcription factors known to regulate the development of PC cNKs include Nfil3, Eomes, and to a lesser extent, T-bet (52). In contrast, PC helper ILC1 development is critically dependent on T-bet, but not Eomes or Nfil3 (52). Additionally, both PC cNKs and helper ILC1s develop in a Hobit-independent manner, unlike liver helper ILC1s (52).

### Other Tissues

In addition to the populations described above, increasing evidence suggests that tissue-resident NK and/or helper ILC1s do or may exist in many other tissues. For example, in addition to CD49b^+^CD49a^−^ cNK-like cells, the kidney contains a minor subset (15–20%) of CD49a^+^CD49b^−^ cells that are tissue-resident (114). These trNK and/or helper ILC1s are TRAIL^+^CD44^hi^, but express little CD62L or KLRG1. Importantly, this tissue-resident population(s) was specifically associated with tissue damage following ischemic acute kidney injury, suggesting a unique pathogenic role for these cells (114).

CD49a^−^CD49b^+^ cNK and CD49a^+^CD49b^−^ trNK (called ILC1-like in the study) populations have also been described in the mammary glands (MG) (115). Both subsets are CD127^−^T-bet^+^, but MG cNK cells are Eomes^hi^, whereas MG trNKs are Eomes^lo^ (115). In addition, a portion of the MG trNKs, but few or none of the MG cNKs, express CD103, Ly49E, and TRAIL (115). Functionally, both MG populations were poor producers of IFN-γ and TNF-α, but remain capable of killing tumor cells through a perforin-dependent pathway (115).

The lacrimal gland (LG) also contains several distinct populations of Eomes^+^ NK and Eomes^−^ helper ILCs with still undefined tissue-residency properties (116). CD49b^+^CD49a^−^ cells comprise the majority of the LG compartment, with CD49b^+^CD49a^+^ and CD49b^−^CD49a^+^ cells representing minority populations. Similarly, the majority of cells in the LG compartment are CD27^+^KLRG1^low^TRAIL^−^CD127^−^ and are functionally hyporesponsive, producing less IFN-γ than splenic cNKs during viral infection (116). However, LG cells that have been adoptively transferred into lymphocyte-deficient hosts and then recovered from the spleen and liver, are no longer hyporesponsive (116). These findings suggest that the altered functionality of LG NKs and/or helper ILC1s may be instructed by the LG tissue environment, analogous to the effect of the SG environment on SG NKs.

Additionally, a distinct population of NKp46^+^CD3^−^ cells has been shown to localize to the exocrine pancreas in young non-obese diabetic (NOD) mice and to infiltrate the endocrine pancreas in adult NOD mice, possibly reflecting a role in diabetes-related autoimmunity (117). In NOD mice, this pancreatic population is generally hyporesponsive to stimulation, exhibiting reduced IFN-γ production and CD107a upregulation following receptor crosslinking, but displays higher spontaneous production of IFN-γ *ex vivo*, as compared to splenic cNKs (117). Phenotypically, these cells are CD69^hi^CD27^hi^KLRG1^+^, but express little or no CD62L and CD127 (117). Later studies established that most are CD49b^+^, although a small number are CD49a^+^ (64). Whether the CD49b^+^ or CD49a^+^ subsets are comprised of *bona fide* tissue-resident NKs and/or helper ILC1s remains to be determined.

Overall, relatively little is known about the specific developmental requirements of kidney, MG, LG, and pancreatic NK cells, with a few exceptions. cNKs in the MG, bulk CD3^−^NK1.1^+^ cells in the LG, and the non-tissue-resident CD49b^+^CD49^−^ cNK-like subset in the kidney are all significantly reduced in Nfil3-mice, suggesting that development of these populations is fully or partially Nfil3-dependent (114–116). In contrast, MG trNKs are not significantly reduced in mice lacking Nfil3, and the tissue-resident CD49a^+^CD49b^−^ NK and/or ILC1 subset in the kidney is actually more abundant (115, 116). Notably, T-bet-deficiency does not significantly alter the number of CD49a^+^CD49b^−^ or CD49a^−^CD49b^+^ cells in the kidney (114).

## Concluding Remarks

Recent studies have demonstrated that, like many other immune cell lineages, unique tissue-resident NK and helper ILC1 populations exist in a broad array of tissues and organs. These populations exhibit tissue-specific phenotypes and functions, and have important roles in diverse biological processes with both positive and negative consequences for organismal health. Examples include the immunomodulatory function of SG trNKs in dampening T cell-mediated tissue damage in the SG during viral infection, and the negative impact of IFN-γ-producing NK and helper ILC1 populations in the adipose tissue on obesity-related metabolic dysfunction (102, 106–110). As discussed above, some but not all trNKs and helper ILC1s share expression of markers associated with tissue-residency, e.g., CD49a, CD103, CD200r1, and CD69, although the phenotype and functional properties of these populations are often unique to the tissue environment in which they exist.

**Figure 1:**
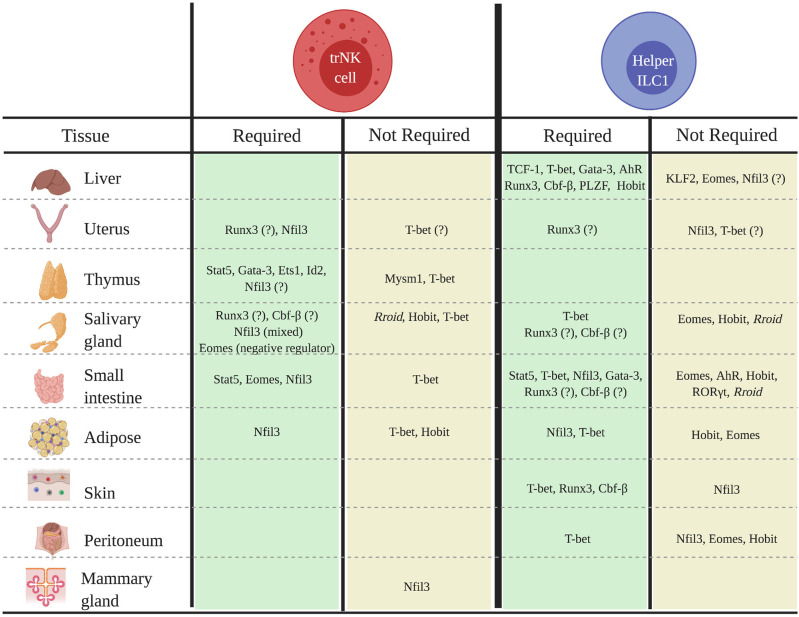
Transcriptional regulators of the development of tissue-specific trNK and helper ILC1 populations in mice. Schematic shows known transcriptional regulators of trNK or helper ILC1 development or differentiation in specified tissues.

While our understanding of the transcriptional networks that regulate tissue-specific trNKs and helper ILC1s is still very limited, the balance of evidence suggests that these populations often exhibit unique developmental requirements that can differ not only across tissues, but also among the cNK, trNK, and helper ILC1 populations in a specific tissue (Figure 1). Indeed, findings that some trNKs and helper ILC1s in the liver, uterus, skin, kidney, salivary gland, and mammary glands do not strictly require Nfil3, and that trNKs and/or helper ILC1s in the kidney, uterus, and adipose tissue do not require T-bet for development, are particularly notable, given the key roles of these factors in the development of cNKs and other ILC subsets. Understanding why some, but not all, trNK and helper ILC1 populations bypass developmental requirements for otherwise “lineage-defining” factors such Nfil3 and/or T-bet is an important topic for future studies.

Although this review has focused principally on studies involving tissue-resident NKs and helper ILC1s in mice, it is important to note that similar tissue-associated populations have now been described in diverse human tissues, including the uterus, adipose, tonsils, intestines, liver, kidney, and lung (118, 119). Most of these tissue-associated populations are CD56^bright^, distinguishing them from highly cytotoxic CD56^dim^ cNKs in peripheral blood, although accurate discrimination of human trNKs and helper ILC1s is currently limited by the lack of faithful lineage-tracking markers and tools (120). Nevertheless, these tissue-associated CD56^bright^ populations as a whole share many similarities with mouse trNKs and helper ILC1s, including high expression of tissue residency-associated surface markers (e.g., CD69, CD49a, and CD103) and chemokine receptors (e.g., CXCR6 and CCR5), and low or no expression of CD62L and CCR7 (121). For example, the human liver harbors a population of CD56^bright^ cells that are Eomes^high^ and express CD49a, CXCR6, and CD69 (122). These CD56^bright^ cells are long-lived (up to 13 years) and persist in the liver without recirculation, suggesting they likely represent *bona fide* liver trNKs/ILC1s (122). Notably, these cells also express Hobit, raising the possibility that Hobit may regulate liver trNK/ILC1 development in humans, similar to its role in mice (123). Although, little is currently known about transcriptional regulation of human trNK/ILC1 development, a better understanding of these pathways could inform the development of novel therapies to treat or prevent human disease.

It is now appreciated that NK cells are capable of mediating adaptive immune responses in certain settings. Indeed, memory or memory-like responses have been described for tissue-resident NKs and helper ILC1s in the liver, skin, and uterus (60, 62, 112, 124). Whether trNKs in other tissues are also capable of immunological memory, and the impact such responses might have on tissue homeostasis and chronic inflammatory diseases, remains to be elucidated. Other important and outstanding questions in the field include: how do tissue-resident vs. bone marrow-derived progenitors contribute to the replenishment of tissue-specific trNKs at steady state and during inflammation? How do tissue-specific signals shape the phenotype and function of trNKs? And finally, will the use of single-cell technologies—e.g., scRNA-seq—reveal new and previously unappreciated heterogeneity in the trNK populations that exist within various tissues? Addressing these questions will critically shape our understanding of the unique biological processes that regulate trNK and helper ILC1 biology at steady state and in settings of tissue-specific inflammatory diseases.

## Author Contributions

NV-P and AB wrote the manuscript and designed the figure.

### Conflict of Interest

The authors declare that the research was conducted in the absence of any commercial or financial relationships that could be construed as a potential conflict of interest.
