# Corrigendum: Transcriptional Regulation of Mouse Tissue-Resident Natural Killer Cell Development

**DOI:** 10.3389/fimmu.2020.01355

**Published:** 2020-07-08

**Authors:** Nuriban Valero-Pacheco, Aimee M. Beaulieu

**Affiliations:** ^1^Center for Immunity and Inflammation, New Jersey Medical School, Rutgers Biomedical and Health Sciences, Rutgers–The State University of New Jersey, Newark, NJ, United States; ^2^Department of Microbiology, Biochemistry, and Molecular Genetics, New Jersey Medical School, Rutgers Biomedical and Health Sciences, Rutgers–The State University of New Jersey, Newark, NJ, United States

**Keywords:** natural killer cells, tissue-resident NK cells, transcriptional regulation, transcription factors, group 1 innate lymphoid cells

In the original article, there was an error. Several words were omitted in the first sentence of a paragraph which altered the meaning of a sentence. A correction has been made to the section the **Development of Tissue-Specific or Tissue-Resident NK Cells and Helper ILC1s**, subsection **Liver**, paragraph 2. The corrected paragraph appears below:

“Phenotypically, liver ILC1s resemble immature cNKs in having low or no expression of killer cell lectin-like receptor G1 (KLRG1), CD11b, CD122, and Ly49 receptors such as Ly49A, Ly49D, Ly49G2, and Ly49H (50, 51, 63, 64). However, liver ILC1s are transcriptomically distinct from both immature and mature cNKs and exhibit an activated phenotype at steady state, characterized by high expression of CD69, CD44, and CD160, and low expression of CD62L (also known as L-selectin) (51, 59, 64, 65). They also express high levels of tumor necrosis factor (TNF)-related apoptosis-inducing ligand (TRAIL) and CD127, as well as chemokine receptors such as CXCR3 and CXCR6 that support residence in the liver sinusoids (50, 51, 59, 64, 66, 67). Although activated liver ILC1s retain cytotoxic functionality against target cells, they differ from cNKs in their higher production of TNF-α, IL-2, and granulocyte-macrophage colony-stimulating factor (GM-CSF), their preferential expression of granzyme C instead of granzyme B, and their reduced expression of perforin (51, 59, 64, 65). Liver ILC1s also express molecules involved in immune regulation, including PD-L1, LAG3, CD39, and CD73, and were recently shown to inhibit T cell function via the PD-1–PD-L1 axis (68).”

The authors apologize for this error and state that this does not change the key scientific conclusions of the article in any way. The original article has been updated.

